# Disinfection of human cardiac valve allografts in tissue banking: systematic review report

**DOI:** 10.1007/s10561-016-9570-9

**Published:** 2016-08-13

**Authors:** M. Germain, D. M. Strong, G. Dowling, J. Mohr, A. Duong, A. Garibaldi, N. Simunovic, O. R. Ayeni, on behalf of the Bioburden Steering Committee and Cardiac Tissue Working group, Amber Appleby, Scott Brubaker, Jeannie Callum, Graeme Dowling, Ted Eastlund, Margaret Fearon, Marc Germain, Cynthia Johnston, Ken Lotherington, Ken McTaggart, Jim Mohr, Jutta Preiksaitis, Michael Strong, Martell Winters, Kimberly Young, Jie Zhao, Graeme Dowling, Marc Germain, Sonny Lazaro, Jim Mohr, Michael Strong, Jacynthe Tremblay

**Affiliations:** 1 Héma-Québec, 1070 Sciences-de-la-Vie Avenue, Quebec, QC G1V 5C3 Canada; 2 Department of Orthopaedics and Sports Medicine, University of Washington School of Medicine, 98104 Seattle, WA USA; 3 Comprehensive Tissue Centre, 8230 Aberhart Centre, 11402 University Avenue NW, Edmonton, AB T6G 2J3 Canada; 4 Canadian Blood Services, 270 John Savage Ave., Dartmouth, NS B3B 0H7 Canada; 5 Department of Surgery, McMaster University, 293 Wellington St. N, Suite 110, Hamilton, ON L8L 8E7 Canada; 6 McMaster University Medical Centre, 1200 Main St W, Room 4E15, Hamilton, ON L8N 3Z5 Canada

**Keywords:** Cardiovascular allografts, Tissue donation, Tissue decontamination, Bioburden, Tissue banking

## Abstract

Cardiovascular allografts are usually disinfected using antibiotics, but protocols vary significantly between tissue banks. It is likely that different disinfection protocols will not have the same level of efficacy; they may also have varying effects on the structural integrity of the tissue, which could lead to significant differences in terms of clinical outcome in recipients. Ideally, a disinfection protocol should achieve the greatest bioburden reduction with the lowest possible impact on tissue integrity. We conducted a systematic review of methods applied to disinfect cardiovascular tissues. The use of multiple broad spectrum antibiotics in conjunction with an antifungal agent resulted in the greatest reduction in bioburden. Antibiotic incubation periods were limited to less than 24 h, and most protocols incubated tissues at 4 °C, however one study demonstrated a greater reduction of microbial load at 37 °C. None of the reviewed studies looked at the impact of these disinfection protocols on the risk of infection or any other clinical outcome in recipients.

**Electronic supplementary material:**

The online version of this article (doi:10.1007/s10561-016-9570-9) contains supplementary material, which is available to authorized users.

## Introduction

Prior to the advent of tissue preservation, transplantation of cardiac valves had to occur shortly after recovery to reduce the incidence of contamination and tissue damage. Advances in cardiovascular preservation have allowed for the creation of heart valve banks worldwide to increase the number and quality of heart valves available for transplantation (Chaukar et al. 1990; Gall et al. 1998; Germain et al. 2010; Goffin et al. 1996; Heng et al. 2013a, b; Jashari et al. 2007; Tabaku et al. 2004; Verghese et al. 2004; Villalba et al. 2009). However, additional processing steps have increased the prevalence of contamination in these tissues. The presence of microorganisms can pose a serious and sometimes lethal threat to the transplant recipient (CDC, C. for D. C. and P 1997; Kuehnert et al. 1998). Heart valve banks have employed a variety of procedures to both determine and reduce bioburden, which has improved the quality of stored grafts, as well as the outcomes for the transplant recipient (Tabaku et al. 2004).

The *contamination rate* represents the proportion of tissues with bacterial or fungal contamination, and the *bioburden* denotes the quantity of organisms on each sample. As it relates to cardiac grafts, bioburden reduction (disinfection) is defined as a process applied following recovery, which reduces or eliminates bacteria or fungal contamination (Kairiyama et al. 2009). Reduction due to antimicrobial intervention can be assessed qualitatively in relation to changes in contamination rate or quantitatively by determining the bioburden load before and after an intervention. Secondary outcomes, following disinfection, will allow the assessment of the effects of bioburden reduction processes on tissue viability and structural integrity.

## Methods

### Information sources and search

The search strategy was developed and reviewed by the Cardiac Processing and Validation Subgroup (through JM) and assisted by an information specialist. The search was applied to electronic databases MEDLINE and EMBASE from 1988 to July 2, 2014 using the following headings and text words: “heart valve,” “cardiac valve,” “aortic valve,” “pulmonary valve,” “allograft,” “anti-bacterial,” “anti-fungal,” “sterilization,” and “tissue banking.” The search included publications in English and excluded animal studies, case reports and conference abstracts. Two additional reviewers (AG and AD) performed a second search to include publications from July 2014 up to March 6, 2015. The detailed search strategy is shown in Online Resource 1.

### Study selection

Three reviewers (CP, JM, and SF) independently screened each of the citations in duplicate to identify studies that included an evaluation of disinfection of human cardiac valves or cardiac conduits, and/or included bioburden as an outcome. If during the screening process there were disagreements, the full report was retrieved and the independent assessment was repeated. Disagreements for inclusion were resolved by consensus.

### Data abstraction

The design of data abstraction forms and evidence tables were guided by the questions in the analytic framework (Online Resource 2) and approved and finalized by the Cardiac Processing and Validation Subgroup (through JM). Two reviewers (AG and AD) independently collected the data, and a third reviewer (NS) confirmed the data abstraction for the following study characteristics: first author, year of publication, location of study, sample size, donor types, recovery site, tissue types, pre-recovery skin preparation, storage and incubation parameters, and preservation methods. Microbial testing methods were documented for each study. Abstracted outcome data included: bioburden immediately following recovery, antimicrobial intervention following bioburden assessment, incubation parameters, proportion of allografts discarded due to contamination, and logarithmic reduction of bioburden load.

### Quality assessment

There were no clinical studies found among the final pool of included articles and therefore no studies that could be qualified by the GRADE assessment. There is no validated quality assessment tool for laboratory-based studies, such as GRADE, because basic science research is inherently considered level IV, or low quality evidence (Guyatt et al. 2011).

### Data analysis

Data abstracted from all of the included studies were organized into tables demonstrating study characteristics, microbial testing methods, and outcomes. Descriptive statistics included the bioburden outcome, the proportion of discarded allografts and the logarithmic reduction of bioburden. Proportions, means, ranges, and measures of variance such as standard deviations (SD) are presented when available. Where appropriate, data analysis was performed separately for the report by Heng et al. (2013a), as this study monitored bioburden reduction among 24 different sites internationally (Heng et al. 2013a).

## Results

### Study selection

**Fig. 1:**
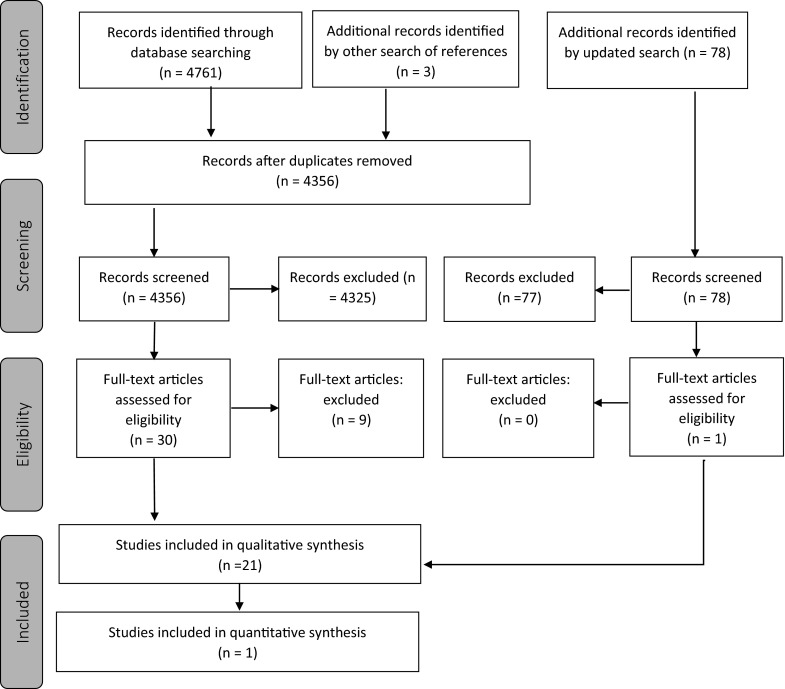
Screening process flow diagram

A total of 4353 citations were reviewed after duplicates were removed and three additional citations were identified by a separate search of references (Fig. 1). Of the 4356 citations, 4325 were excluded because they did not fulfill the screening criteria. The full text-articles of the remaining 31 citations were retrieved for further evaluation. Twenty-one laboratory based studies that reported a disinfection method and bioburden as an outcome were included. Nine studies were excluded for varying reasons listed in Online Resource 3. Following the updated search, an additional 78 articles were reviewed, and one was identified for further evaluation. The article was included in this review.

### Study characteristics and culture methods

All included studies were conducted from 1990 to 2013. Six of the 22 laboratory investigations (Table 1) were conducted in Belgium; two each from Australia, India, Singapore, and Spain; and one each from Bangladesh, Brazil, Canada, Ireland, Netherlands, South Africa, and the USA. One additional study was conducted at 24 different sites worldwide (Heng et al. 2013a). Six studies indicated that the recovery of tissues from organ donors was performed in operating theatres of a hospital setting. Five studies recovered cadaveric tissues in the autopsy room. Thirteen studies did not specify where recovery took place (Online Resource 4).

**Table 1:** Study characteristics

First author, year	Country	Sample size	Donors	Recovery site	Tissues
Heng et al. (2013b)	Singapore	57 heart valves from 28 donors	Cadaveric living	Operating theatre	Cardio-vascular allografts
Heng et al. (2013a)	Europe (11)	1620 heart valves 858 donors	NR	NR	Cardio-vascular allografts
	North America (6)	11,095 heart valves 3831 donors			
	Australasia and South Africa (7)	542 heart valves 267 donors			
Villalba et al. (2012)	Spain	849 heart valves	NR	Operating theatre	Cardio-vascular allografts
Botes et al. (2012)	South Africa	2540 heart valves 1792 donors	Cadaveric	NR	Cardio-vascular allografts
Fan et al. (2012)	Belgium	814 heart valves 241 arteries	Cadaveric living	NR	Cardio-vascular allografts
Heng et al. (2012)	Singapore	NR	NR	NR	NR
Soo et al. (2011)	Ireland	564 heart valves 297 donors	Cadaveric living	Operating theatre	Cardio-vascular allograft
van Kats et al. (2010)	Netherlands	376 heart valves 275 donors	Cadaveric living	NR	Cardio-vascular allograft
Jashari et al. (2010)	Belgium	5133 heart valves 2066 arteries	Cadaveric living organ	Operating theatre	Cardio-vascular allografts
Germain et al. (2010)	Canada	NR	NR	NR	Cardio-vascular allografts
Villalba et al. (2009)	Spain	1412 heart valves	NR	NR	Cardio-vascular allografts
Jashari et al. (2007)	Belgium	184 heart valves and arteries 80 heart donors	Cadaveric living organ	NR for living and organ donors	Cardio-vascular allografts
				Autopsy room (cadaveric donors)	
Hoque et al. (2007)	Bangladesh	30 heart valves	Cadaveric	NR	Cardio-vascular allografts
Peruzzo et al. (2006)	Brazil	1671 heart valves	NR	NR	Cardio-vascular allografts
Ireland and Spelman (2005)	Australia	857 heart valves	NR	NR	Musculoskeletal skin and cardiac allografts
Tabaku et al. (2004)	Belgium	956 heart valves 541 donors	Cadaveric living organ	Operating theatre (living and organ donors)	Cardio-vascular allografts
				Autopsy room (cadaveric donors)	
Verghese et al. (2004)	India	790 heart valves 588 donors	Cadaveric living	Autopsy room (cadaveric donors)	Cardio-vascular allografts
Goffin et al. (2000)	Belgium	2478 heart valves 1907 donors	Cadaveric living organ	NR	Homograft heart valves
Goffin et al. (1996)	Belgium	1949 heart valves 981 donors	Cadaveric living organ	NR	Cardio-vascular allografts
Gall et al. (1995)	Australia	642 heart valves	Cadaveric living organ	Operating theatre (living and organ donors)	Cardio-vascular allografts
				Autopsy room (cadaveric donors)	
McNally and Brockbank (1992)	USA	535 heart valves	NR	NR	Cardio-vascular allografts (not suitable for trans-plantation)
Chaukar et al. (1990)	India	206 heart valves	NR	Autopsy room	Cardio-vascular allograft

Following recovery of the cardiac tissue, 7 studies stored the samples in cold saline, and 4 stored the samples in tissue culture medium. The remaining 11 tissue banks did not report their storage solutions. In 10 studies, the reported method of long-term storage of cardiac tissue was through cryopreservation, which is storage in the vapour phase of liquid nitrogen in a cryopreservation medium containing 10 % dimethylsulfoxide (DMSO). Most studies did not report a method for allograft preservation.

The culture methods used to determine bioburden are outlined in Online Resource 5. All studies assessed for the presence of bacteria and fungi in the tissue samples by culturing of a sample of the tissue, and testing for the presence of bacterial or fungal growth. Five studies also included serological analysis for the presence of viruses.

### Study outcomes

#### Microbe identification and bioburden analysis

Microbial sampling was conducted in a total of 33,300 cardiac valves (including arteries) from 7641 donors in 21 studies. Bioburden analysis determined that the most commonly found contaminating bacteria included *Staphylococci, Propionobacterium, Streptococci,* and *Escherichia coli.* In the reports that cultured for fungi, the most predominant fungi were *Candida* species. The contamination rate following recovery of the allografts ranged from 8 to 100 % of the total number of tissue samples isolated (Mean: 31.0 %, SD: 22.7 %) (Online Resource 6). Cryopreservation was utilized for long-term storage of allografts in 14 studies and at all 24 sites in the multinational study. The remaining 9 studies did not indicate a preservation method.

#### Bioburden reduction

Given the relatively high contamination rate following recovery of cardiac tissue, the need to reduce contamination is of utmost importance. In all studies, an antimicrobial-intervention was chosen to reduce contamination. Every study used a combination of broad-spectrum antibiotics, which included but were not limited to, penicillin, streptomycin, cefoxitin, vancomycin, amikacin and gentamicin. The most commonly used antibiotic was vancomycin (77 % of studies). Only 11 of the 22 studies reported the inclusion of an anti-fungal agent (nystatin, polymyxin B or amphotericin B) to reduce fungal bioburden. The greatest reduction in contamination rate was seen in two studies. Villalba et al. (2009) added an antibiotic cocktail composed of amikacin (50 µg/ml), vancomycin (50 µg/ml), metronidazole (50 µg/ml), and amphotericin B 5ug/ml and effectively reduced the number of contaminated tissues to 3.2 %, although the initial number of contaminated tissues was not reported (Villalba et al. 2009). Peruzzo et al. (2006) disinfected  the tissue samples with cefoxitin (240 µg/ml), lincomycin (120 µg/ml), polymyxin B (100 µg/ml), and vancomycin (50 µg/ml), and were able to reduce the proportion of allografts discarded from 8 to 5.6 % (Peruzzo et al. 2006). Both reports included a combination of broad-spectrum antibiotics as well as an anti-fungal agent. The tissues were incubated in the antibiotic solution for 6–24 h at 4 °C, or for 24 h between 2 and 8 °C. Two sites in Europe reported the least effective reduction in contamination rate post-processing (50 %) (Heng et al. 2013a). The reason for failure in site 1 was bacterial contamination, whereas reasons for failure in site 2 included abnormal morphology, bacterial contamination and other technical issues not related to disinfection. Site 1 incubated at 4 °C for 48 h, with the broad-spectrum antibiotic, vancomycin, with the narrow-spectrum antibiotic, lincomycin, and the fungicide, polymyxin B. Site 2 incubated at 2–8 °C for 24 h, with broad-spectrum antibiotic, vancomycin, with gentamicin, imipenem, and the fungicides, nystatin and polymyxin B (Heng et al. 2013a).

In the multi-site study by Heng et al. (2013a), three heart valve banks, of the 24 sites, were able to reduce the proportion of rejected tissues to just 10 % (Heng et al. 2013a). At two sites in Europe, one heart valve bank utilized a combination of vancomycin (50 µg/mL), gentamicin (4000 µg/mL), ciprofloxacin (200 µg/mL), and amphotericin B (50 µg/mL) at 21 °C, while the other bank used fluconazole and cefotaxime to treat heart allografts at 4 °C. The third bank with sites in Australasia or South Africa utilized cefoxitin (240 µg/mL), lincomycin (120 µg/mL), polymyxin B (100 µg/mL), vancomycin (50 µg/mL), and amphotericin B (25 µg/mL) at 4 °C. While the use of multiple broad spectrum antibiotics with an anti-fungal agent was effective in the first European site as well as the Australasian/South African site, the second European bank was able to achieve the same result using only one broad-spectrum antibiotic (cefoxitin) and one anti-fungal agent (fluconazole).

Four studies utilized an incubation temperature of 37 °C instead of 4 °C, and had a reduced incubation time between 6 and 12 h (Gall et al. 1995; Heng et al. 2013b; Ireland and Spelman 2005; van Kats et al. 2010). This resulted in an average reduction of contamination to only 8.1 %, compared to 5.9 % for tissues incubated at 4 °C for an extended period. In the survey by Heng et al. (2013a), there was no difference in the contamination reduction rate between the 6–12 h incubation at 37 °C and 24–48 h incubation at 37 °C (29.8 and 30.2 %, respectively). In Germain et al. (2010), incubation of tissues at 37 °C with antibiotics was more effective in bioburden reduction at higher temperatures. At 37 °C, the authors were able to disinfect the tissues completely, reducing the bioburden from 5000 to 0 CFU/ml (3.7 fold logarithmic reduction in the bioburden). Conversely, incubation of tissues at 4 °C with antibiotics were only able to reduce the bioburden from 5000 to 3.6 CFU/ml (a 3.1 fold logarithmic reduction in bioburden) (Germain et al. 2010).

#### Confounding factors

The use of antibiotics to disinfect the allografts was common among all reports, but the parameters for the antibiotic treatment were highly varied. The time period for which the samples were incubated in the disinfection solution varied from 12 h up to 6 weeks. By extending the incubation period, the authors allowed for elimination of more microorganisms, but viability of the tissues was not assessed following these extended incubation periods. Additionally, four studies reported incubation of the tissue at 37 °C rather than 4 °C as in most other studies (Chaukar et al. 1990; Germain et al. 2010; Heng et al. 2013b; van Kats et al. 2010). At one of the 24 sites in Heng et al. (2013a), the tissue bank modified their incubation temperature from 1–10 °C to 32–38 °C in the recent past (Online Resource 6) (Heng et al. 2013a). The majority of antibiotics are more effective at higher temperatures, but the integrity of the allografts may be compromised at this temperature for extended periods (Goffin et al. 1996).

## Discussion

Following recovery of the heart allograft, the usage of broad and narrow spectrum antibiotics coupled with an antifungal agent at 4 °C for up to 2 days had the greatest reduction in the proportion of allografts contaminated with a microorganism (Villalba et al. 2009). The proportion of cardiac allografts requiring disinfection following recovery was quite high. Cleaning and rinsing methods to reduce bioburden were not reported in these studies, and recovered allografts were often stored in Ringer’s or saline solutions. None of the included studies reported increased implant survival or a reduction in morbidity and mortality following transplantation.

The initial contamination rate was found to be as high as 100 % of all the tissues recovered (Hoque et al. 2007). A method to disinfect the skin prior to recovery of the heart allografts was not reported in most studies. Gall et al. (1995) reported that more allografts were contaminated when being recovered from multi organ donors, as opposed to cadaveric donors (14 vs. 10 %) (Gall et al. 1995). This suggests that further precaution might be required prior to heart valve recovery from multi-organ donors. Although no studies had a control group, two studies demonstrated a lower initial contamination rate of 21.2 % when stored in saline or Ringer’s solution (Goffin et al. 2000; van Kats et al. 2010), compared to exclusive storage of allografts in saline in three other studies (mean contamination rate of 46.4 %) (Fan et al. 2012; Heng et al. 2013b; Hoque et al. 2007).

The most common microorganisms contaminating the allografts are classified as opportunistic pathogens (disease-causing in immunocompromised individuals), and positive culture of any organism warranted disinfection of the allograft. However, in the studies in this review, the researchers disinfected all allografts, regardless of the level of contamination. Fungi, and specifically the genus *Candida* have been identified as significant pathogens that should preclude further transplantation if identified in allografts (CDC, C. for D. C. and P 1997; Kuehnert et al. 1998).

All reports sought to reduce bioburden, using antibiotics. Antibiotics are currently the preferred method of disinfection, as past methods of disinfection using chemicals have been deemed too harsh, resulting in reduced viability of heart valves (Goffin et al. 1996). While most reports did not use the same combination of antimicrobial agents, a lower proportion of allograft discard was associated with studies that used a combination of broad spectrum antibiotics, the narrow spectrum antibiotic, lincomycin, and the antifungal agent, polymyxin B. Heng et al. (2013a) compared the effectiveness of a combination of penicillin and streptomycin (two broad-spectrum antibiotics commonly used for disinfection of allografts) to the use of two other broad-spectrum antibiotics (amikacin and vancomycin). Although incubation parameters varied for each combination, amikacin and vancomycin were capable of reducing the allograft discard rate to 4.7 % (1/21), as opposed to an 11.1 % (4/36) allograft discard rate when penicillin and streptomycin were used (Heng et al. 2013b). It should be noted that the only contaminating organism following treatment with amikacin and vancomycin was a fungus, which was not targeted by these antibiotics. Jashari et al. (2007) reported that the addition of the antifungal agent, polymyxin B to the antibiotic cocktail reduced the proportion of allograft discard to 4.3 %, compared to an allograft discard rate of 5.5 %, when only the antibiotic cocktail (lacking polymyxin B) was added (Jashari et al. 2007). This suggests that fungal contamination could represent a consistent, but minor component of the contaminating bioburden.

The majority of studies reported that the cardiovascular allografts were incubated in an antibiotic-containing solution for 6–24 h at 4 °C. It is hypothesized that lower temperatures allow for the antibiotic to function while maintaining tissue integrity (Villalba et al. 2009). However, in one study, incubation at 37 °C showed a greater reduction in the bioburden load compared to the same treatment at 4 °C (Germain et al. 2010). Extension of the incubation period beyond 24 h does not appear to increase the effectiveness of the antibiotic treatment.

## Limitations

Limitations in this review relate to missing or non-reported data. Following recovery of the allografts, the initial contamination rate was lower when the samples were stored in saline or Ringer’s solution, compared to storing the samples exclusively in saline. The proportion of samples stored in either solution was not reported, and thus, it is indeterminate if the Ringer’s solution could affect the contamination rate following recovery. Also, the addition of cleaning or rinsing agents following recovery to reduce bioburden was not reported in any of the studies, and therefore it remains unclear if these procedures have a significant impact on bioburden reduction.

Another main limitation in this review is the heterogeneity in the experimental design among the primary studies. Variances in initial contamination rate, antibiotic combination and concentration, incubation period, incubation temperature, and assay method to quantify results were rarely tested within studies. Additionally, the standards and regulations regarding the release of contaminated tissues is not universal among tissue banks worldwide. Regulatory organizations like the American Association of Tissue Banks do not allow the release of allografts contaminated with pathogens, but classification of pathogens may differ among countries.

The majority of the articles presented the contamination reduction rate as a proportion of allografts discarded due to contamination or potentially rejected due to positive culture following disinfection. Although this was an effective qualitative metric in terms of quality assessments of the antibiotics’ effectiveness, a more quantitative method, calculating bioburden log reduction, would allow for more informed recommendations for clinical implementation. Reporting of quantified values would allow for optimization of interventions, thus improving the quality of clinical recommendations.

Additionally, certain outcomes, such as tissue viability following antibiotic exposure were not addressed in any of the 22 studies. Tissue viability is principal to cardiovascular transplantation success, and as such, additional studies are required to address this issue. Finally, there was no discussion on the acceptable levels of contamination that would allow for allograft release. Not all organisms would necessarily be pathogenic. Allografts were often discarded based on the presence of positive cultures following the antibiotic intervention, but identification of these remaining organisms (such as the fungal contaminant in Heng et al. 2013a) could elicit additional antimicrobial treatments that could further reduce the proportion of allograft discard.

## Conclusions

The results of this review suggest that the use of multiple broad-spectrum antibiotics in combination with an antifungal agent result in the greatest reduction in bioburden. Antibiotic incubation periods were typically no longer than 24 h, and most samples were incubated at 4 °C. One study showed a greater reduction in microbial load in tissues at 37°C^6^. The majority of studies in this review did not test the efficacies of antimicrobial interventions relative to one another, and were all laboratory studies (level IV evidence). The transplantation of the treated tissues was not performed and evaluated for clinical effectiveness; therefore, these outcomes should be interpreted with caution.

## Electronic supplementary material

Below is the link to the electronic supplementary material.

Supplementary material 1 (PDF 45 kb)

Supplementary material 2 (PDF 14 kb)

Supplementary material 3 (PDF 35 kb)

Supplementary material 4 (PDF 81 kb)

Supplementary material 5 (PDF 80 kb)

Supplementary material 6 (PDF 218 kb)
