# Supplementary material for: Disinfection of human cardiac valve allografts in tissue banking: systematic review report
Source: Cell Tissue Bank. 2016 Aug 13;17(4):593–601. doi: 10.1007/s10561-016-9570-9 (PMC5116039; doi:10.1007/s10561-016-9570-9)
Supplement: Supplementary file 1 — Supplementary material 1 (PDF 45 kb) [file 10561_2016_9570_MOESM1_ESM.pdf]

Online Resource 1: Search Strategy

MEDLINE

1	Transplantation, Homologous/
2	Heart Valves/tr [Transplantation]
3	Aortic Valve/tr [Transplantation]
4	Pulmonary Valve/tr [Transplantation]
5	Aorta/tr [Transplantation]
6	Pulmonary Artery/tr [Transplantation]
7	(transplant* adj2 homologous).mp.
8	((heart valve? or cardiac valve?) adj2 transplant*).mp.
9	((heart valve? or cardiac valve?) adj2 (graft* or isograft* or iso-graft* or allograft* or allo-graft* or autograft* or auto-graft* or autogeneicgraft* or autogeneic-graft* or syngraft* or syn-graft* or syngeneicgraft* or syngeneic-graft* or homograft* or homo-graft*)).mp.
10	((aortic* or aorta*) adj2 transplant*).mp.
11	((aortic* or aorta*) adj2 (graft* or isograft* or iso-graft* or allograft* or allo-graft* or autograft* or auto-graft* or autogeneicgraft* or autogeneic-graft* or syngraft* or syn-graft* or syngeneicgraft* or syngeneic-graft* or homograft* or homo-graft*)).mp.
12	(pulmonary adj2 arter* adj2 transplant*).mp.
13	(pulmonary adj2 arter* adj2 (graft* or isograft* or iso-graft* or allograft* or allo-graft* or autograft* or auto-graft* or autogeneicgraft* or autogeneic-graft* or syngraft* or syn-graft* or syngeneicgraft* or syngeneic-graft* or homograft* or homo-graft*)).mp.
14	(pulmonary adj2 valve? adj2 transplant*).mp.
15	(pulmonary adj2 valve? adj2 (graft* or isograft* or iso-graft* or allograft* or allo-graft* or autograft* or auto-graft* or autogeneicgraft* or autogeneic-graft* or syngraft* or syn-graft* or syngeneicgraft* or syngeneic-graft* or homograft* or homo-graft*)).mp.
16	or/1-15
17	Tissue Preservation/
18	Organ Preservation/
19	"Tissue and Organ Procurement"/
20	Cryopreservation/
21	exp Anti-Bacterial Agents/
22	exp Antifungal Agents/
23	exp Cryoprotective Agents/
24	exp Sterilization/
25	Tissue Banks/
26	((tissue? or organ?) adj2 preserv*).mp.
27	((tissue? and organ?) adj2 procurement*).mp.

28 (cryopreserv* or cryo-preserv*).mp.
29 anti-bacterial agent?.mp.
30 antibacterial agent?.mp.
31 bacteriocid*.mp.
32 antifung* agent?.mp.
33 anti-fung* agent?.mp.
34 fungicid*.mp.
35 (cryoprotective adj2 agent?).mp.
36 (steriliz* or sterilis*).mp.
37 (tissue? adj2 (bank or banking)).mp.
38 (bioburden or bio-burden).mp.
39 or/17-38
40 16 and 39
41 exp animals/ not (exp humans/ and exp animals/)
42 40 not 41
43 limit 42 to (clinical conference or congresses or consensus development conference or consensus development conference, nih)
44 42 not 43
45 limit 44 to (english language and yr="1988 -Current")

EMBASE

1 allotransplantation/
2 aorta graft/
3 (transplant* adj2 homologous).mp.
4 ((heart valve? or cardiac valve?) adj2 transplant*).mp.
5 ((heart valve? or cardiac valve?) adj2 (graft* or isograft* or iso-graft* or allograft* or allo-graft* or autograft* or auto-graft* or autogeneicgraft* or autogeneic-graft* or syngraft* or syn-graft* or syngeneicgraft* or syngeneic-graft* or homograft* or homo-graft*)).mp.
6 ((aortic* or aorta*) adj2 transplant*).mp.
7 ((aortic* or aorta*) adj2 (graft* or isograft* or iso-graft* or allograft* or allo-graft* or autograft* or auto-graft* or autogeneicgraft* or autogeneic-graft* or syngraft* or syn-graft* or syngeneicgraft* or syngeneic-graft* or homograft* or homo-graft*)).mp.
8 (pulmonary adj2 arter* adj2 transplant*).mp.
9 (pulmonary adj2 arter* adj2 (graft* or isograft* or iso-graft* or allograft* or allo-graft* or autograft* or auto-graft* or autogeneicgraft* or autogeneic-graft* or syngraft* or syn-graft* or syngeneicgraft* or syngeneic-graft* or homograft* or homo-graft*)).mp.
10 (pulmonary adj2 valve? adj2 transplant*).mp.

11	(pulmonary adj2 valve? adj2 (graft* or isograft* or iso-graft* or allograft* or allo-graft* or autograft* or auto-graft* or autogeneicgraft* or autogeneic-graft* or syngraft* or syn-graft* or syngeneicgraft* or syngeneic-graft* or homograft* or homo-graft*)).mp.
12	or/1-11
13	tissue preservation/
14	organ preservation/
15	Cryopreservation/
16	exp antifungal agent/
17	cryoprotective agent/
18	((tissue? or organ?) adj2 preserv*).mp.
19	((tissue? and organ?) adj2 procurement*).mp.
20	(cryopreserv* or cryo-preserv*).mp.
21	anti-bacterial agent?.mp.
22	antibacterial agent?.mp.
23	bacteriocid*.mp.
24	antifung* agent?.mp.
25	anti-fung* agent?.mp.
26	fungicid*.mp.
27	(cryoprotective adj2 agent?).mp.
28	(steriliz* or sterilis*).mp.
29	(tissue? adj2 (bank or banking)).mp.
30	(bioburden or bio-burden).mp.
31	or/13-30
32	12 and 31
33	(exp animals/ or exp animal experimentation/) not ((exp animals/ or exp animal experimentation/) and exp human/)
34	32 not 33
35	limit 34 to (conference abstract or conference paper or conference proceeding or "conference review")
36	34 not 35
37	limit 36 to (english language and yr="1988 -Current")
38	limit 37 to embase
