# Supplementary material for: Disinfection of human cardiac valve allografts in tissue banking: systematic review report
Source: Cell Tissue Bank. 2016 Aug 13;17(4):593–601. doi: 10.1007/s10561-016-9570-9 (PMC5116039; doi:10.1007/s10561-016-9570-9)
Supplement: Supplementary file 2 — Supplementary material 2 (PDF 14 kb) [file 10561_2016_9570_MOESM2_ESM.pdf]

Online Resource 2: Analytical Framework

Cardiac Processing and Validation

1. What heart valve processing method, or combination of methods, will be the most effective in reducing and controlling tissue bioburden (quantitative load) while maintaining tissue quality (the ability of the tissue to function as intended)?
    a. What are the advantages and disadvantages of each method?
        i. Ischemic time limits?
        ii. Antibiotic selection?
        iii. Antibiotic incubation?
        iv. Cleaning and rinsing?
        v. Storage and transportation?

    b. What is the maximum ischemic time limit?
        i. Does ischemic time correlate to bioburden content (load?)

    c. What antibiotic / antifungal combination is optimal for reducing the bioburden content? Optimal is defined as the highest log reduction in bioburden. A six log reduction is considered sterile.
        i. What incubation parameters maximize the reduction of bioburden content (load)?
            1. Temperature?
            2. Duration?
            3. What should be the basis for establishing these parameters?
                a. Are there industry standards?
                b. What evidence supports this impact?

    d. What cleaning and rinsing processes are most effective in reducing bioburden content (load)?
        i. Rinsing solution?
        ii. Rinsing parameters?
        iii. Testing for pyogenes?

    e. What storage and transportation parameters are most effective in reducing bioburden content (load)?
        i. Storage methodology?
        ii. Storage temperature?
        iii. Transportation process?
2. In cardiovascular patients for cardiac valve implantation, does human cardiac valve allograft (HCVA) processed on a specific method or combination result in increased implant survival (leaflet failure) and/or reduction in mortality/infection/co-morbidities?

3. What is the prevalence of cardiovascular patients in need of HCVA implant?
    a. What proportion of patients receives an HCVA implant to increase survival and decrease morbidities?
    b. Are there patients at risk of failure or success of transplant? Risks refer to relevant patient data?
