# Supplementary material for: Disinfection of human cardiac valve allografts in tissue banking: systematic review report
Source: Cell Tissue Bank. 2016 Aug 13;17(4):593–601. doi: 10.1007/s10561-016-9570-9 (PMC5116039; doi:10.1007/s10561-016-9570-9)
Supplement: Supplementary file 3 — Supplementary material 3 (PDF 35 kb) [file 10561_2016_9570_MOESM3_ESM.pdf]

Online Resource 3: Excluded Studies

Studies	Reasons for Exclusion
Bisdas, 2009	No cardiac valves, all vascular tissues, did not determine which antibiotic regimen or heart valve processing method more effective in reducing and controlling tissue bioburden
Blem, 2013	Case report
Center for Disease Control, 1997	Investigation of a case report
Hickey, 2007	20-30 year follow up of graft survival in patients
Johnson, 1998	Effect of antibiotic on immunogenicity
Kowalski, 2012	Microbial sampling of the bone
Leeming, 2005	Assay method to determine antibiotic concentration
Warwick, 2008	Survey of tissue banking
Yap, 2004	Review article on valve replacement in adult

Bisdas, T. E., Mattner, F., Ott, E., Pichlmaier, M. A., Wilhelmi, M., Haverich, A., & Teebken, O. (2009). Significance of infection markers and microbiological findings during tissue processing of cryopreserved arterial homografts for the early postoperative course. Vasa, 38(4), 365-373.

Blehm, A., Schurr, P., & Lichtenberg, A. (2013). Role of primary bacterial contamination of a pulmonary homograft for Ross operation: report of a case and review of the literature. The Thoracic and cardiovascular surgeon, 61(6), 541-542.

Center for Disease Control (1997) Candida albicans Endocarditis Associated with a Contaminated Aortic Valve Allograft -- California, 1996, Morbidity and Mortality Weekly Report, 46(12);261-263

Hickey, E., Langley, S. M., Allemby-Smith, O., Livesey, S. A., & Monro, J. L. (2007). Subcoronary allograft aortic valve replacement: parametric risk-hazard outcome analysis to a minimum of 20 years. The Annals of thoracic surgery,84(5), 1564-1570.

Johnson, D. L., Sloan, C., O'Halloran, A., & Yacoub, M. H. (1998). Effect of antibiotic pre-treatment on immunogenicity of human heart valves and component cells. The Annals of thoracic surgery, 66(6), S221-S224.

Kowalski, J. B., Merritt, K., Gocke, D., & Osborne, J. (2012). Assessment of bioburden on human and animal tissues: Part 2—Results of testing of human tissue and qualification of a composite sample for routine bioburden determination. Cell and tissue banking, 13(3), 431-439.

Leeming, J. P., Lovering, A. M., & Hunt, C. J. (2005). Residual antibiotics in allograft heart valve tissue samples following antibiotic disinfection. Journal of Hospital Infection, 60(3), 231-234.

Pitt, T. L., Tidey, K., Roy, A., Ancliff, S., Lomas, R., & McDonald, C. P. (2014). Activity of four antimicrobial cocktails for tissue allograft decontamination against bacteria and Candida spp. of known susceptibility at different temperatures. Cell and tissue banking, 15(1), 119-125.

Warwick, R. M., Magee, J. G., Leeming, J. P., Graham, J. C., Hannan, M. M., Chadwick, M., ... & Parker, R. (2008). Mycobacteria and allograft heart valve banking: an international survey. Journal of Hospital Infection, 68(3), 255-261.

Yap, C. H., & Yii, M. (2004). Allograft aortic valve replacement in the adult: a review. Heart, Lung and Circulation, 13(1), 41-51.
