# Supplementary material for: Disinfection of human cardiac valve allografts in tissue banking: systematic review report
Source: Cell Tissue Bank. 2016 Aug 13;17(4):593–601. doi: 10.1007/s10561-016-9570-9 (PMC5116039; doi:10.1007/s10561-016-9570-9)
Supplement: Supplementary file 4 — Supplementary material 4 (PDF 81 kb) [file 10561_2016_9570_MOESM4_ESM.pdf]

Online Resource 4: Allograft Recovery Methods

.

First Author, Year	Pre-Recovery Skin Preparation	Post-Recovery Storage conditions	Preservation Method
Heng, 2013 (1)	NR	Following recovery, storage in cold saline (2-8˚C)	Cryopreservation<br>Preserved in 10% DMSO in medium 199 (M199) and temperature was reduced -1˚C per minute until -50˚C
Heng, 2013 (2)	NR	NR	Cryopreservation<br>Controlled rate freezing
		NR	Cryopreservation<br>Controlled rate freezing
		NR	Cryopreservation<br>Controlled rate freezing
Villalba, 2012	NR	NR	Cryopreservation
Botes, 2012	NR	NR	Cryopreservation<br>Preserved in 11% DMSO in medium 199 (M199) and temperature was reduced -1˚C per minute until -140˚C
Fan, 2012	NR	Following recovery, storage in cold saline (2-8˚C)	Cryopreservation<br>Preserved in 10% DMSO in medium 199 (M199) and temperature was reduced -1˚C per minute until -150˚C to -187˚C
Heng, 2012	NR	NR	NR
Soo, 2011	NR	Following recovery, storage in cold saline (2-8˚C) then Medium 199 with Hanks' Salts and antibiotics	Cryopreservation<br>Preserved in 10% DMSO in RPMI1640 medium and temperature was reduced: from 4 to 0°C at a rate of -5°C per minute; From 0 to -15°C at a rate of -1°C per minute; From -15 to -16°C at a rate of -0.1°C per minute; From -16 to -80°C at a rate of -1°C per minute; From -80 to -

First Author, Year	Pre-Recovery Skin Preparation	Post-Recovery Storage conditions	Preservation Method
			140°C at a rate of -5°C per minute and from -140 to -180°C at a rate of -1°C per minute
Van Kats, 2010	NR	Tissue was transferred to cardioplegia, saline or Ringer's solution for short term storage.	Cryopreservation<br>Preserved in cryoprotective medium at <-150˚C
Jashari, 2010	NR	Following recovery, storage in saline 4˚C.	Cryopreservation<br>Preserved in 10% DMSO in medium 199 (M199) and temperature was reduced -1˚C per minute until -40˚C and then -5˚C per minute until -100˚C. Tissues are permanently stored at <-150˚C.
Germain, 2010	NR	NR but tissue extracted from tissue bank	NR
Villalba, 2009	NR	NR	Cryopreservation<br>Preserved in 10% DMSO in cryoprotective medium and temperature was reduced -1˚C per minute until -60˚C and then -5˚C per minute until -120˚C. Tissues are permanently stored at -150˚C
Jashari, 2007	NR	Following recovery, storage in saline, ringer or medium 199 with Hanks Salts and L-Glutamine (Medium 199) at +4 ^{o}C.	NR
Hoque, 2007	NR	Following recovery, storage in cold saline (4˚C)	NR
Peruzzo, 2005	NR	Following recovery, storage in cold saline (2-8 ^{o}C)	NR
Ireland, 2005	Skin prep by shaving then two 5 min	NR	Cryopreservation<br>Storage in vapour phase of nitrogen

First Author, Year	Pre-Recovery Skin Preparation	Post-Recovery Storage conditions	Preservation Method
	surgical scrubs using chlorhexidine solution		
Tabaku, 2004	NR	NR	Cryopreservation
Verghese 2004	NR	Following recovery, storage in Hank's balanced salt solution (HBSS) at 4˚C.	Cryopreservation<br>Preserved in 10% DMSO in media 199 (M199), reducing the temperature by 1˚C per minute for 90 minutes, followed by storage in liquid nitrogen (-120˚C to -160˚C)
Goffin, 2000	Either cold TCM 199, saline, Ringer, Eurocollins, UW	Following recovery, storage in saline, Ringer, Eurocollins, UW, or tissue culture medium 199 in HEPES buffer (TCM 199).	Cryopreservation<br>Preserved in 10% DMSO in Tissue culture medium 199 (TCM199) and incubated at 4˚C for 40 – 60 min, and then temperature is reduced 1˚C per minute until -40˚C, and then by -5˚C per minute until -100˚C.
Goffin, 1996	NR	NR	Cryopreservation<br>Preserved in 10% DMSO in Tissue culture medium 199 (TCM199) and incubated at 4˚C for 40 – 60 min, and then temperature is reduced 1˚C per minute until -40˚C, and then by -5˚C per minute until -100˚C.
Gall, 1995	NR	Following recovery, storage in medium 199 (M199) on wet ice.	Cryopreservation<br>Preserved in 10% DMSO in M199 and temperature was reduced -1˚C per minute until -40˚C and then stored in vapour phase of liquid nitrogen
McNally, 1992	NR	NR	Cryopreservation
Chaukar, 1990	NR	Following recovery, stored in Hank's balanced salt solution (HBSS)	NR

NR = not reported; organ = multi organ donor; living = receipt of heart transplant
