# Supplementary material for: Disinfection of human cardiac valve allografts in tissue banking: systematic review report
Source: Cell Tissue Bank. 2016 Aug 13;17(4):593–601. doi: 10.1007/s10561-016-9570-9 (PMC5116039; doi:10.1007/s10561-016-9570-9)
Supplement: Supplementary file 5 — Supplementary material 5 (PDF 80 kb) [file 10561_2016_9570_MOESM5_ESM.pdf]

Online Resource 5 - Culture Methods

First Author, Year	Identified organisms	Microbial Testing Method
Heng, 2013 (1)	Viruses<br>Bacteria<br>Fungi	Serological analysis of antibodies for Hepatitis B antigen and Human T-Lymphotrophic virus (HTLV). PCR analysis for Dengue flavivirus. *Bacillus* acid fast test for presence of *Mycobacterium tuberuculosis*. Bacteria and fungi were identified by microbiological cultures.
Heng, 2013 (2)	NR	NR
Villalba, 2012	Bacteria<br>Fungi	Samples were incubated in thioglycollate fluid medium, Trypticase soy broth and Sabouraud dextrose chloramphenicol agar for at least 14 days.
Botes, 2012	Bacteria<br>Fungi<br>Viruses	Microbial culture for aerobes, anaerobes, fungi and mycobacteria were performed to test for contamination. Serological testing for Hepatitis B, Hepatitis C, HIV, HTLV and polymerase chain reaction analysis for IV were also done.
Fan, 2012	Bacteria<br>Fungi<br>Viruses	Microbial culture for aerobes, anaerobes and fungi were performed to test for contamination.
Heng, 2012	Bacteria<br>Fungi<br>Viruses	Aerobic bacteria were cultured on both blood agar and MacConkey agar plates and incubated in 5% carbon dioxide at 35°C for 48 h. Anaerobic bacteria were cultured on CDC anaerobic blood plates, and incubated in an anaerobic chamber at 35°C for 96 h. Fungi were inoculated onto SDA and incubated at 30°C for 4 wks.
Soo, 2011	Bacteria<br>Fungi	Tissue samples are inoculated Heart Infusion (BHI), Sabaroud and Thioglycollate broths and cultured for 6 days. After day 6, the broths are subcultured for another 3 days if a positive culture is seen. Serological analysis is performed for HIV, Hepatitis C, Hepatitis B, HTLV and syphilis.
Van Kats, 2010	Bacteria	Tissue samples were inoculated into the appropriate medium for aerobic (chocolate) and anaerobic (Schaedler) bacteria, as well as Brewer's broth and Brain—Heart Infusion (BHI) broth. After anti-microbial treatment, the samples were tested by inoculation into BHI broth and thioglycolate broth and grown for 7 days at 37˚C.
Jashari, 2010	Viruses<br>Bacteria<br>Fungi	Serological analysis for hepatits B, C, HIV, HTLV, syphilis, malaria, viral myocarditis, enteroviruses, or active tuberculosis. Bacteriologic examination for aerobic and anaerobic bacteria and fungi.
Germain, 2010	Bacteria	Microorganisms were inoculated into trypticase soy broth or thioglycollate medium at 22±2˚C and 37±2˚C, respectively, for up to 14 days in aerobic or anaerobic conditions.
Villalba, 2009	Bacteria	NR, but tested for presence of aerobic and anaerobic bacteria and fungi

First Author, Year	Identified organisms	Microbial Testing Method
Jashari, 2007	Bacteria Fungi	Aerobic and anaerobic bacteria are cultured in thioglycolate medium, enriched with a resazurine, 37°C for 14 days and fungal test with Sabouraud culture medium enriched with trypto-caseine and soja, 37°C for 14 days. Mycobacteria is cultured in Lowenstein-Jansen medium for 7 weeks.
Hoque, 2007	Bacteria Fungi	Bacterial cultures were grown in thioglycollate broth at 37˚C. Fungi were cultured in Sabouraud dextrose broth at 27˚C for 6 weeks.
Peruzzo, 2005	Bacteria Fungi	Cultures were grown in thioglycolate (Thio) medium at 35˚C for 14 days for growing aerobic and anaerobic bacteria, trypticase in soy broth (TSB) at 35˚C for 14 days for the growing of aerobic bacteria and Sabouraud broth at 22˚C for 14 days for the growing of fungi. For Thio and TSB cultures with positive cultures, secondary cultures were performed in MacConkey agar to isolate Gram negative bacteria and sheep blood agar to isolate (Gram positive and negative) media.
Ireland, 2005	Bacteria	Swabs of bone and tissue samples, as well as cardiac and allograft skin tissues were placed into thioglycollate medium, incubated for 48 h at 35°C.The medium was sub-cultured onto Columbia blood agar plates, and incubated aerobically and anaerobically at 35°C for up to seven days.
Tabaku, 2004	Bacteria Fungi	Microbiologic analyses for aerobic bacteria, anaerobic bacteria and fungal contamination were carried out during 14 days on two enriched culture media.
Verghese, 2004	Bacteria Fungi	Tissue and rinse solutions were cultured in nutrient broth, thioglycollate broth, Robertson’s cooked medium (RCM), and Sabouraud’s dextrose broth at two temperatures. Positive cultures were subcultured on blood agar, MacConkey agar, or Sabouraud’s dextrose agar.
Goffin, 2000		Culturing for aerobic bacteria, anaerobic bacteria, and fungi were used. Serologic analysis for HIV1, HIV2, HTLV, HCV, VDRL and PCR analysis for HIV1 was performed.
Goffin, 1996	Bacteria Fungi	Culturing for aerobic bacteria, anaerobic bacteria, and fungi were used. Serologic analysis for HIV1, HIV2, HTLV, HCV, VDRL and PCR analysis for HIV1 was performed.
Gall, 1995	Bacteria Fungi	Tissue samples were inoculated into nutrient broth and thioglycolate broth and cultured at 35˚C for 48 hours. The nutrient broth was subcultured and incubated aerobically, where the thioglycolate broth was grown anaerobically at 35˚C for an additional 48 hours. The wash and storage solutions were plated on horse blood agar at 35˚C for 48 hours. The rinse and wash solutions were cultured on Sabouraud dextrose agar at 30ºC for 21 days.
McNally, 1992	Bacteria Fungi	NR
Chaukar, 1990	Bacteria Fungi	Tissues were inoculated in nutrient broth, thioglycollate broth and sabouraud's broth. Samples were monitored at intervals of 24 h, 48 h, 1 wk, 2 wk, 3 wk, 4 wk, 5 wk and 6 wk.

BHI= Brain—Heart Infusion; DNA= deoxyribonucleic acid; ELISA= enzyme-linked immunosorbent assay; HCV= Hepatitis C virus; HTLV1= Human T-Lymphotrophic virus 1; NR = not reported; PCR= polymerase chain reaction; RCM= Robertson's cooked medium; SD= Sabouraud dextrose agar; TSB= trypticase soy broth; THIO= thioglycolate; VDRL= Venereal Disease Research Laboratory
