# Supplementary material for: Disinfection of human cardiac valve allografts in tissue banking: systematic review report
Source: Cell Tissue Bank. 2016 Aug 13;17(4):593–601. doi: 10.1007/s10561-016-9570-9 (PMC5116039; doi:10.1007/s10561-016-9570-9)
Supplement: Supplementary file 6 — Supplementary material 6 (PDF 218 kb) [file 10561_2016_9570_MOESM6_ESM.pdf]

Online Resource 6 - Study Outcomes

First Author, Year	Microbes Detected Immediately following recovery	Contamination Rate Immediately Following Recovery	Antimicrobial intervention Following Bioburden Assessment	Incubation Parameters	Proportion of allografts discarded due to contamination	Bioburden Reduction
Heng, 2013a	*Propionibacterium acnes*<br>*Pseudomonas spp.*<br>*Staphylococcus*<br>*Escherichia coli*<br>*Acinetobacter spp.*<br>*Micrococcus spp*<br>Methicillin-resistant *Staphylococcus aureus (MRSA)*<br>*Rhodococcus*<br>*Streptococcus*<br>*Bacillus spp.*<br>*Candida spp.*	13.9% Positive culture (5/36)	Penicillin G 50 IU/mL , Streptomycin 50 µg/mL	37°C for between 6 to 12 h	11.1% of grafts discarded after treatment (4/36)<br><br>2 bacteria<br>1 fungi<br>1 MRSA	NR
		42.9% Positive culture (9/21)	Amikacin and vancomycin (concentration NR)	4°C for 24 – 48 h	4.7% of grafts discarded (1/21) Positive fungal culture	NR
Heng, 2013b	NR	NR (Europe)	Vancomycin: 500 µg/ml<br>Gentamycin: 50 µg/ml<br>Piperacillin: 500 µg/ml<br>Nystatin: 2500 U/mL	Room temperature , 24 hours, in the dark	21% Discarded	NR
			Cefoxitin: 240 µg/ml<br>Lincomycin: 120 µg/ml<br>Colimycin: 100 µg/ml<br>Vancomycin: 50 µg/ml	4°C, 24 hours	30% Discarded	NR
			Gentamicin: 4000 µg/ml<br>Imipenem: 200 µg/ml<br>Nystatin: 2500 U/mL<br>Polymyxin B: 200 µg/ml	2–8°C, 18–24 hours	50% Discarded	NR

First Author, Year	Microbes Detected Immediately following recovery	Contamination Rate Immediately Following Recovery	Antimicrobial intervention Following Bioburden Assessment	Incubation Parameters	Proportion of allografts discarded due to contamination	Bioburden Reduction
			Vancomycin: 50 μg/ml			
			Vancomycin: 50 μg/ml<br>Gentamicin: 4000 μg/ml<br>Ciprofloxacin: 200 μg/ml<br>Amphotericin B: 50 μg/ml	Room temperature (21°C), 24 NR hours	10% Discarded	NR
			Metronidazol: 50 μg/ml<br>Vancomycin: 50 μg/ml<br>Amikacin: 50 μg/ml<br>Amphotericin B: 5 μg/ml	4°C NR, 24 hours	31% Discarded	NR
			Vancomycin: 50 μg/ml<br>Tobramycin: 50 μg/ml<br>Cotrimoxazole: 50 μg/ml	4°C, 6–24 hours	30% Discarded	NR
			Fluconazole: 200mg<br>Cefotaxime: 1 g	4°C, 24 hours	10% Discarded	NR
			Metronidazole: 20 μg/ml<br>Gentamicin: 20 μg/ml<br>Flucloxacillin: 20 μg/ml	4°C, at a minim NR NR um of 12 hours	20% Discarded	NR
			Lincomycin, polymyxin B sulphate, vancomycin	4°C, 48 hours	50% Discarded	NR
			Amphotericin B: 250 μg/ml<br>Fungoral: 100 μg/ml<br>Colistin: 200 μg/ml	4°C, 24 hours	32% Discarded	NR

First Author, Year	Microbes Detected Immediately following recovery	Contamination Rate Immediately Following Recovery	Antimicrobial intervention Following Bioburden Assessment	Incubation Parameters	Proportion of allografts discarded due to contamination	Bioburden Reduction
			Vancocin: 500 µg/ml<br>Garamycin: 530 µg/ml			
			Cefuroxime: 250 µg/ml<br>Gentamicin: 80 µg/ml<br>Ciprofloxacin: 200 µg/ml<br>Vancomycin: 500 µg/ml<br>Colistin: 1000 IU/mL<br>Amphotericin B: 20 µg/ml	37°C, 18–24 hours	22% Discarded	NR
		NR (North America)	Vancomycin: 50 µg/ml<br>Gentamicin: 80 µg/ml<br>Cefoxitin: 240 µg/ml	Until June 28,2010: 1∘C–10∘C, 22–26 hours<br>Present: 33∘C–38∘C, 18–26 Hours	NR	NR
			Cefoxitin, colymycin-M, vancomycin, lincomycin	4∘C, 24 hours	30% Discarded	NR
			Gentamicin: 80mg/mL<br>Cefazolin or Kefzol: 1mg/mL	4∘C, 24 hours	22% Discarded	NR
			Cefoxitin: 240 µg/ml	1∘C–10∘C, 22–26	40% Discarded	NR

First Author, Year	Microbes Detected Immediately following recovery	Contamination Rate Immediately Following Recovery	Antimicrobial intervention Following Bioburden Assessment	Incubation Parameters	Proportion of allografts discarded due to contamination	Bioburden Reduction
			Polymyxin B: 100 mg/mL<br>Vancomycin: 50 µg/ml<br>Lincomycin: 120 µg/ml	hours		
			NR	Warm solutions, >24 hours	61% Discarded	NR
			Vancomycin: 50 µg/ml<br>Colymycin M: 75mg/mL<br>Cefoxitin: 100mg/mL<br>Lincomycin: 300mg/mL	4∘C, 24 ± 2 hours	NR	NR
		NR (Australasia and South Africa)	Amoxicillin: 20 µg/ml<br>Gentamicin: 20 µg/ml	37∘C, 6–8 hours or 4∘C, 18–24 hours	25% Discarded	NR
			Penicillin: 50 IU/mL<br>Streptomycin: 50 µg/ml	35∘C, 6–8 hours	50% Discarded	NR
			Penicillin: 50 IU/mL<br>Streptomycin: 50 µg/ml	37∘C, 6–12 hours	30% Discarded	NR
			Benzylpenicillin: 30 µg/ml<br>Gentamicin: 18 µg/ml	37∘C, 6 hours	17% Discarded	NR
			Cefoxitin: 240 µg/ml<br>Lincomycin: 120 µg/ml<br>Polymyxin B: 100 µg/ml<br>Vancomycin: 50 µg/ml	First soak: 4∘C, 24 hours;<br>2nd soak: 4∘C, 24	10% Discarded	NR

First Author, Year	Microbes Detected Immediately following recovery	Contamination Rate Immediately Following Recovery	Antimicrobial intervention Following Bioburden Assessment	Incubation Parameters	Proportion of allografts discarded due to contamination	Bioburden Reduction
			Amphotericin B: 25 µg/ml	hours; transfer to HBSS at 4∘C until frozen		
			Vancomycin: 50 µg/ml Amikacin: 100 µg/ml	4∘C, 24 hours	29% Discarded	NR
			Mefoxin: 50 µg/ml Piperacillin: 50 µg/ml Amikacin: 25 µg/ml Amphotericin B: 2.5 µg/ml	4∘C, 12–18 hours	39% Discarded	NR
Villalba, 2012	High virulence<br>*Enterococcus faecalis Enterobacter spp. Staphylococcus aureus Escherichia coli Klebsiella pneumoniae Proteus vulgaris Pseudomonas aeruginosa*<br><br>Low virulence<br>*Staphylococcus negative coagulase Corynebacterium spp. Streptococcus spp.*	10.8% Positive culture (92/849)	Vancomycin (50 µg/ml) Tobramycin (50 µg/ml) Cotrimoxazole (50 µg/ml)	Incubated between 6 and 24 h at 4°C	NR	NR

First Author, Year	Microbes Detected Immediately following recovery	Contamination Rate Immediately Following Recovery	Antimicrobial intervention Following Bioburden Assessment	Incubation Parameters	Proportion of allografts discarded due to contamination	Bioburden Reduction
Botes, 2012	HIV<br>Hepatitis B<br>Venereal disease<br>*Mycobacterium tuberculosis*	NR	Mefoxin sodium 50 mg<br>Piperacillin 50 mg<br>Amikacin 25 mg<br>Amphotericin B 2.5 mg	4°C for 6-8 wks	39.2% of grafts discarded (995/2540)<br>32.4% HIV<br>9.6% Hep B<br>5.6% positive cultured organism	NR
Fan, 2012	*Staphylococcus spp,*<br>*Escherichia coli,*<br>*Propionibacterium spp.*<br>*Klebsiella spp.*<br>*Candida spp.*<br>*Corynbacteria*<br>Gram positive cocci<br>*Pseudomonas*<br>*Streptococcus*	25.3% Positive culture (206/814)	Lincomycin HCL 120 µg/ml<br>Polymyxib B Sulphate 124 µg/ml<br>Vancomycin HCL 50 µg/ml	4°C for between 20 and 48 h	19.9% Potentially discarded , but NR (41/206 positive cultures following decontamination)	NR
Heng, 2012	*Pseudomonas spp.*<br>*Staphylococcus spp.*<br>*Escherichia coli*<br>*Rhodococcus*<br>*Propionbacteria spp*<br>*Bacillus spp*<br>*Aspergillus spp*<br>*Candida spp*	13.9% positive culture (5/36)	Penicillin<br>Streptomycin	NR	NR, but >0%<br><br>1 positive culture for MRSA discarded after antimicrobial treatment	NR
		33.3% positive culture (11/33)	Amikacin<br>Vancomycin	NR	9.09% of grafts discarded<br><br>3/33 Fungal contaminants after	NR

First Author, Year	Microbes Detected Immediately following recovery	Contamination Rate Immediately Following Recovery	Antimicrobial intervention Following Bioburden Assessment	Incubation Parameters	Proportion of allografts discarded due to contamination	Bioburden Reduction
					antimicrobial treatment	
Soo, 2011	*Mycobacterium cheloneii* atypical acid fast *Bacillus Candida famata.*	NR	Gentamicin<br>Metronidazole<br>Flucloxacillin	4ºC for at least 12 h	20.7% of grafts discarded (117/564)	NR
Van Kats, 2010	<u>High virulence</u><br>*Streptococcus spp.*<br>*Escherichia coli*<br>*Staphylococcus aureus*<br>*Haemophilus spp.*<br>*Clostridium spp.*<br>*Enterobacter spp. Serratia spp. Aeromonas spp. Bacteroid spp.*<br><br><u>Low virulence</u><br>Coagulase-negative *Staphylococcus Bacillus spp. Propionibacterium spp.*<br>*Corynebacterium spp*<br>*Flavimonas spp*	17% Positive culture (63/376)	Cirpofloxacin 3 µg/ml<br>Amikacin 12 µg / ml<br>Vancomycin 12 µg /ml<br>Metronidazole 12 µg /ml<br>Flucytosine 30 µg /ml	37°C for 5-6 h.	18% positive culture (67/376)	NR
Jashari, 2010	NR	NR	Vancomycin<br>Lincomycin<br>Polymyxin B	20-48 h	NR	

First Author, Year	Microbes Detected Immediately following recovery	Contamination Rate Immediately Following Recovery	Antimicrobial intervention Following Bioburden Assessment	Incubation Parameters	Proportion of allografts discarded due to contamination	Bioburden Reduction
Germain, 2010	*Propionibacterium acnes Staphylococcus aureus Staphylococcus epidermidis Streptococcus salivarius Escherichia coli Pseudomonas aeruginosa Bacillus subtilis Klebsiella pneumoniae Streptococcus agalactiae Lactococcus lactis*<br>*Bacillus spp.*	Typical, but NR in this study<br><br>Aerobic bacteria<br>52 ± 212 CFU/ml<br><br>Aerobic and anaerobic bacteria<br>77 ± 245 CFU/ml	Vancomycin 50 µg/ml, Gentamicin 80 µg/ml, Cefoxitin 240 µg/ml	4 ± 2°C for 24 ± 2 h (Cardiac tissue)	NR	Bacterial count reduced from 5000 CFU/ml to 3.6 CFU/ml
				37 ± 2°C for 24 ± 2 h (Cardiac tissue)	NR	Bacterial count reduced from 5000 CFU/ml to 0 CFU/ml
Villalba, 2009	*Enterococcus faecalis, Rhodororula, Bacillus spp, Aspergillus spp Staphylococcus epidermidis*	NR (Tissue Bank A)	Vancomycin (50 µg/ml) Tobramycin (50 µg/ml) Cotrimoxazole (50 µg/ml) Amphotericin B (50 µg/ml)	Incubated between 6 and 24 h 3at 4°C	3.2% of allografts discarded overall (6/189)<br><br>2.64% positive culture (5/189)	NR
		NR (Tissue Bank B)	Vancomycin (50 µg/ml) Tobramycin (50 µg/ml) Cotrimoxazole (50 µg/ml) Amphotericin B (50 µg/ml)	Incubated between 6 and 24 h at 4°C	1.05% positive culture (1/95)	NR
		NR (Tissue Bank C)	Amikacine (50 µg/ml) Vancomycin (50 µg/ml) Metronidazole (50 µg/ml)	Incubated between 6 and 24 h at 4°C	0% positive culture (0/20)	NR

First Author, Year	Microbes Detected Immediately following recovery	Contamination Rate Immediately Following Recovery	Antimicrobial intervention Following Bioburden Assessment	Incubation Parameters	Proportion of allografts discarded due to contamination	Bioburden Reduction
			Amphotericin B 5µg/ml			
Jashari, 2007	*Staphylococci Citrobacter Escherichia coli Pseudomonas Streptococci*	23.75% positive culture (19/80)	Cefoxitin 240 µg/ml<br>Lincomycin 120 µg/ml<br>Polymixiyne B 100 µg/ml<br>Vancomycin 50 µg/ml	4˚C for 20-48 h	5.5% had positive culture after treatment (10/182)	NR
			Lincomycin 120 µg/ml<br>Polymixiyne B 100 µg/ml<br>Vancomycin 50 µg/ml	4˚C for 20-48 h	4.3% had positive culture after treatment (8/184)	NR
Hoque, 2007	*Staphylococcus aureus*<br>*Candida albicans*<br>*Bacillus spp*<br>*Streptococcus pyogenes*<br>*Proteus mirabilis*<br>*Pseudomonas aeruginosa*<br>*Klebsiella pneumonia*	100% positive culture (30/30)	Ceftriaxone 250 µg/l, Lincomycin 120 µg/l, Polymyxin B 100 µg/l, Vancomycin 50 µg/l and Amphotericin B 25 µg/l	48 h	30% had positive culture after treatment (9/30)	NR
Peruzzo, 2005	*Rhodotorula* spp.<br>*Bacillus diphtheroids,*<br>*Acinetobacter lwoffi*<br>*Candida albicans*<br>*Pseudomonas spp.*<br>*Eschericia coli*	8% positive culture (136/1671)<br>17/136	Cefoxitin 240 µg/ml<br>Lincomycin 120 µg/ml<br>Polymycin B 100 µg/ml<br>Vancomycin 50 µg/ml	2-8˚C for 24 h	NR<br><br>5.66% had positive culture after antibiotic treatment (87/1535)	NR

First Author, Year	Microbes Detected Immediately following recovery	Contamination Rate Immediately Following Recovery	Antimicrobial intervention Following Bioburden Assessment	Incubation Parameters	Proportion of allografts discarded due to contamination	Bioburden Reduction
	Staphylococcus epidermidis<br>*Staphylococcus aureus*<br>*Pseudomonas spp*.<br>*Candida albicans*					
Ireland, 2005	Pathogen<br>*Clostridium perfringens*<br><br>Commensal (Non pathogenic)<br>*Staphylococci*<br>*Diphtheroids*<br>*S. aureus*<br>*Bacillus spp*<br>*Propionibacterium acnes*<br>Yeast	15.7 % (84/534) Positive cultures<br><br>85% (72/84) positive cultures (normal skin bacteria)<br><br>10.7% (9/84) Positive samples *Clostridium perfringens* (8/9 isolated from one donor)	Streptomycin (50 µg/ml)<br>Penicillin (30 µg/ml)	35°C for 6–8 h	NR	NR
Tabaku, 2004	Coagulase negative *Staphylococcus*,<br>*Escherichia coli*<br>*Streptococcus viridans*	36.4% positive culture (348/956)	Cefoxitin (0.528 µg/ ml)<br>Lincomycin (0.048 µg/ml)<br>Polymyxin B (0.2 µg/ml)	4˚C for 20-72 h	6% of allografts discarded (57/899)	NR

First Author, Year	Microbes Detected Immediately following recovery	Contamination Rate Immediately Following Recovery	Antimicrobial intervention Following Bioburden Assessment	Incubation Parameters	Proportion of allografts discarded due to contamination	Bioburden Reduction
			Vancomycin (0.05 µg/ml)			
Verghese, 2004	*Klebsiella, Enterobacter, Escherichia coli, Citrobacter, Pseudomonas, Aeromonas, Proteus, Flavobacterium, Diptheroids, Staphylococcus spp. Streptococci, Streptomyces Micrococcus, Clostridium, Candida*	NR for bacteria<br><br>7.4% positive serological result for hepatitis B, hepatitis C, and HIV (44/588)	Amikacin (100 µg/ml)<br>Gentamycin (120 µg/ml)<br>Cefotaxime (250 µg/ml)<br>Amphotericin B (25µg/ml)<br>Vancomycin (50 µg/ml)<br><br>Since 2001 Gentamycin replaced with Streptomycin (115µg/ml)	Incubated at 4°C for 24 hours, and transferred to new storage solution with antibiotics every 24 hours for 3 days	3.9% of allografts discarded due to bacterial contamination (23/588)	NR
Goffin, 2000	NR	25.4% positive culture (441/1739)<br><br>5.85% positive result for viruses (145/2478)	Cefoxitin 240 µg/ml<br>lincomycin 120 µg/ml<br>Polymyxin B 100 µg/ml<br>Vancomycin 50 µg/ml	4"C for 24 h for recovery in sterile conditions 4˚C for 48 hours if donor was kept on respirator for > 24 h	NR<br><br>5.7% positive culture after antibiotic treatment (100/1739)	NR

First Author, Year	Microbes Detected Immediately following recovery	Contamination Rate Immediately Following Recovery	Antimicrobial intervention Following Bioburden Assessment	Incubation Parameters	Proportion of allografts discarded due to contamination	Bioburden Reduction
Goffin, 1996	NR (mentions bacteria and fungi)<br><br>Hepatitis B<br>Hepatitis C	15.91% positive culture (155/974)<br><br>5% positive results for viruses (48/959)	Cefoxitin 240 µg/ml<br>Lincomycin 120 µg/ml<br>Polymyxin B 100 µg/ml<br>Vancomycin 50 µg/ml	4ºC for 24 h	NR<br><br>4.4% positive culture after antibiotics (43/974)	NR
			Cefoxitin 240 µg/ml<br>Lincomycin 120 µg/ml<br>Polymyxin B 100 µg/ml<br>Vancomycin 50 µg/ml<br>Nystatin 2,500 IU/ml added if procured under clean conditions and/or if donor was kept on respirator for > 24 h	4ºC for 48 h		NR
Gall, 1995	*Staphylococcus epidermidis*<br>*Viridans streptococcus*<br>*Streptococcus spp.*<br>*Staphylococcus aureus*<br>*Bacillus spp.*<br>*Viridans streptococcus*<br>*Pseudomonas spp.*	54% positive culture (84/339)	Penicillin 30 µg/ml<br>Streptomycin 50 µg/ml	37ºC for 6 h	5% of allografts discarded (31/642)	
McNally, 1992	NR	NR	Cefoxitin, Polymyxin B, Vancomycin, Lincomycin Dose NR	4ºC for 24 h	14% of allografts discarded (57/406)	NR

First Author, Year	Microbes Detected Immediately following recovery	Contamination Rate Immediately Following Recovery	Antimicrobial intervention Following Bioburden Assessment	Incubation Parameters	Proportion of allografts discarded due to contamination	Bioburden Reduction
	Yeast (primarily *Candida*)<br>*Streptococcus viridans*<br>*Propionibacterium acnes*<br>*Enterococcus* spp.<br>*Staphylococcus* spp.<br>*Bijidobacterium* spp.<br>Saprophytic fungi	51.5% positive culture (275/534) after initial antibiotic treatment (above)	Cefoxitin, Vancomycin, Lincomycin, Cefotaxime Netilmicin, Rifampin, Dose NR	37°C	(Mostly fungal due to lack of antifungal treatment)	NR
Chaukar, 1990	*Klebsiella,*<br>*Pseudomonas*<br>*Proteus*<br>*Escherichia coli*<br>*Bacillus subtillis*<br>*Staphylococci*<br>*Aspergillus*<br>*Mucor*<br>*Penicillium*	NR	Benzylpenicillin 2,000 U/ml<br>Gentamicin 800 µg/ml<br>Hamycin 78 µg/ml	Up to 6 weeks	NR<br>8.6% positive culture after antibiotic treatment(5/58)	NR
			Benzylpenicillin 2000 U/ml<br>Gentamicin 1 mg/ml<br>Hamycin 62.5 µg/ml	Up to 6 weeks	NR<br>18.3% positive culture after antibiotic treatment (27/147)	NR

NR = Not reported
